# Bioactive compounds from *Cucumis melo* L. fruits as potential nutraceutical food ingredients and juice processing using membrane technology

**DOI:** 10.1002/fsn3.2888

**Published:** 2022-04-24

**Authors:** Sana Mallek‐Ayadi, Neila Bahloul, Semia Baklouti, Nabil Kechaou

**Affiliations:** ^1^ Research Group of Agri‐Food Processing Engineering Laboratory of Applied Fluids Mechanics, Process Engineering and Environment National School of Engineers of Sfax University of Sfax Tunisia; ^2^ Laboratory of Materials Engineering and Environment National School of Engineers of Sfax University of Sfax Tunisia

**Keywords:** bioactive compounds, clarification process, melon (*Cucumis melo* L.) pulp, membrane technology, volatile profile

## Abstract

The present study was designed to evaluate the nutritional composition of melon pulp Maazoun variety, in order to explore its potential attitude as a natural source of nutrients and bioactive molecules. The chemical characterization showed that the pulp was rich in moisture, carbohydrate, dietary fibers, and minerals, as well as carotenoids and phenolic compounds. The chromatographic analysis indicated that amentoflavone (16.14 mg/100 g) and gallic acid (13.56 mg/100 g) were the most abundant phenolic compounds. Melon flesh has an interesting volatile profile in which, mostly esters and alcohols are considered as the key odorants of this appreciated fruit. Melon juice was filtered through crossflow microfiltration that provides more translucent juice and accentuation of yellow color. During clarification process, the permeate flux was reduced by 50% in approximately 40 min. Results proved that the richness of melons in nutrients and bioactive phytochemicals makes them useful as a potential source of antioxidants and suitable as nutraceutical supplements.

## Graphical abstract

Melon pulp contains nutrients such as minerals, fibers, carotenoids, and vitamin C. Maazoun flesh could be interesting as a dietary supplement in food applications. The high‐performance liquid chromatography analysis indicated that amentoflavone and gallic acid were the major phenolic compounds. Melon pulp is a natural source of bioactive compounds with nutritive value and antioxidant effects. Clarification was illustrated by a more translucent juice and accentuation of yellow color.

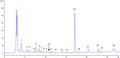

## 1 INTRODUCTION

Nowadays, there is a new trend about the consumption of fresh fruits and vegetables which become more appealing to consumers as an appetizer (soups, salads, and desserts) with delicious taste. Furthermore, fruit and vegetable juices have a high nutritional value, since they are enriched with minerals, vitamins, and other beneficial components for human health (Farcuh et al., 2020; Liu et al., 2019). Compared with other food crops, fruits provide a major source of bioactive molecules. Diets rich in natural biologically active compounds like phenolics and carotenoids are associated with a reduced risk of cancers, cardiovascular diseases, and macular degeneration (Ismail et al., 2010; Silva et al., 2018; Stahl & Sies, 2005). For these reasons, fruit consumption has recently become a concern of health due to growing recognition of its nutritional and therapeutic value.

In this respect, muskmelons (*Cucumis melo* L.) are an important cultivated food crop grown in the tropical and temperate regions of the world whose global production was estimated to more than 40 million tons (FAOSTAT, 2019). With numerous identified varieties, melons have a tough rind and watery flesh with seeds embedded in it. Moreover, they are different in shape, size, rind and pulp colors, and flesh flavor. Melon fruits are highly considered as desserts for their unique aromatic flavor. The widespread growing of melons is related to their desirable sensory attributes. Actually, melon pulp is also used to prepare processed products such as jelly and refreshing juices and drinks. In addition to its excellent flavor qualities, the fruit pulp has a rich nutritional composition and it is an important source of bioactive substances for humans (Petkova & Antova, 2015). Likewise, melons possess a high nutritive value because the pulp provides vitamins and a number of nutrients like protein and folic acid in the daily dietary (Ismail et al., 2010). Therefore, the acceptance of melons is related not only to its juicy pulp considered as fresh and tasty with pleasant aroma, but also to its potential human health benefits.

Traditionally, melons have been used for medicinal reasons so as to relieve constipation due to their dietary fiber, water richness, and nutrient contents (Kolayli et al., 2010). They are also used against gastritis, malnutrition, and bronchial problems. Consequently, it seems that the phytochemicals' quantification in the edible part of melon is of utmost importance to substantiate their potential health benefits in human nutrition. On the other hand, some juices have unfortunately, after their preparation, a cloudy appearance often undesired by consumers as was the case of melon juice. In order to minimize the turbidity of the *C. melo* juice while maintaining its organoleptic and sensory characteristics, a clarification step becomes necessary as an industrial juice processing. Besides, scarce scientific papers are present in the literature about phenolic compounds and volatile profile of melons; therefore, it was required to perform researches on bioactive compounds of fruit pulp and to investigate their nutritional value.

Thus, the present study aims to assess some physicochemical characteristics and bioactive compounds of melons from the Maazoun variety, and to propose microfiltration of melon juice, since such a process is considered as an indicator of food quality. These findings would allow to exploit the potentiality of melon fruits as a source of healthy compounds and could serve as the basis for suggesting changes in the industrial processing of fruits.

## 2 MATERIALS AND METHODS

### 2.1 Raw material

Fully ripened melons (*C. melo* L.) belonging to “Maazoun” cultivar were obtained from plants in the same locality, Skhira (Sfax, Tunisia) (geographical location: latitude 38°94′N; longitude 9°21′E). Melons were inspected carefully for bruising and compression damage and fruit with no visual defects were selected in order to obtain homogeneous samples. Immediately after arrival at the laboratory, melon fruits were washed with running tap water and then cut by hand in longitudinal sections. The skin was uniformly removed using a disinfected stainless steel knife, the blossom and stem ends were discarded, and the placental tissue and seeds were removed. At the same time, the fruit slices were cut into small cubes which were prepared from a number of fruits and randomized before analyses. Besides, a number of melon pulp cubes were crushed with a blender (Moulinex) and the homogenized flesh or juice was used to determine the total soluble solids (TSS) content, pH, titratable acidity (TA), volatile compounds and for clarification process.

All reagents were of analytical grade. Standards (purity ˃ 99.0%) and chemicals were supplied from Sigma‐Aldrich Ltd and from Merck.

### 2.2 Chemical composition of melon pulp

Moisture, protein, fat, and ashes were obtained according to Association of Official Agricultural Chemists (AOAC) (2002). Dry matter was determined by drying samples at 105 °C to constant weight. Protein was estimated according to the Kjeldahl method. The protein content was calculated using a conversion factor (*N* × 6.25). The fat content was analyzed gravimetrically by following Soxhlet extraction with hexane as solvent. Data were expressed as the percentage of fresh weight. Ash was determined by combustion of the sample in a muffle furnace for 4 h, at about 550°C. Ashes were expressed as the percentage of fresh weight. The residue was dissolved in nitric acid (HNO_3_) and the mineral constituents (K, Mg, Ca, Na, Fe, Zn, Mn, and Cu) were analyzed separately, using an atomic absorption spectrophotometer (Analytikjena AAS Zeenit 700, Germany) (AOAC, 1984). The carbohydrate content was estimated by seeking the difference of mean values: 100 − (sum of percentages of moisture, ash, protein, and fat) (Lima et al., 2014). Total dietary fiber was determined according to the AOAC enzymatic–gravimetric method of (Prosky et al., 1988). Fibers were expressed as the percentage of fresh weight.

### 2.3 Total soluble solids content, pH, and titratable acidity determinations

Total soluble solids content was determined using a digital handheld refractometer (Atago PR‐101). Calibration was completed by deionized water and the lens was washed between diverse essays. Values were expressed as Brix degrees (°Brix). The pH was measured at 20 °C with a digital portable electrode pH‐meter (MP 220; Mettler‐Toledo GmbH). The pH electrode and temperature probe were rinsed with distilled water before immersing them in samples. Titratable acidity values were obtained according to the Official Methods of Analysis (AOAC, 2002). Results were expressed as grams citric acid per 100 g melon.

### 2.4 Fruit color and firmness

The surface color was measured on the fresh‐cut pulp using a portable colorimeter Chroma‐meter (CR‐410; Konica Minolta, Inc.) based on CIE L* a* b* color space, with a CIE D65 standard illuminant. For the firmness evaluation, the fruit was divided into two parts along the longitudinal axis and the flesh firmness was conducted using a penetrometer mounted on a Texture Analyzer (Lloyd Instruments). Measurements were carried out on four opposite sides in the equatorial mesocarp tissue. A cylindrical probe (8‐mm diameter) was used to puncture the flat surface of the melon samples at a constant speed of 1.0 mm/s to a deformation of 5 mm. Firmness parameter was calculated from the deformation curve as maximum force using the manufacturer's software. Flesh color and firmness measurements were not made on skin‐facing or seed cavity sides of the flesh cubes.

### 2.5 Measurement of vitamin C

The vitamin C content was determined by titration using 2,6‐dichlorophenolindophenol (DCPIP, 0.05%, w/v; Sigma–Aldrich Chemie) (AOAC, 2002). Briefly, 10 g of melon pulp was homogenized with 40 ml of 3% cold metaphosphoric acid using a kitchen blender (Moulinex). The mixture was filtered with cotton wool. Then, 5 ml of the aliquot was titrated with DCPIP solution until a weak pink color was observed. Results were expressed as milligrams per 100 g fresh weight.

### 2.6 Determination of carotenoid content

Total carotenoids were extracted according to the method of Talcott and Howard (1999), with slight modifications. Two grams of homogenized pulp was extracted using 25 ml of acetone/ethanol (1:1, v/v) with butylated hydroxytoluene (200 mg/L). All manipulations were carried out under a yellow fluorescent light to avoid light‐induced changes. After extraction, the sample was centrifuged (Hettich Zentrifugen, ROTINA 380R), at 1500 × *g* for 15 min at 4°C. Subsequently, the supernatant was collected and the remaining residue was re‐extracted using the same method until the residue was colorless. Finally, the supernatants were made up to 100 ml with the extraction solvent and the absorbance at 470 nm was read by a spectrophotometer (Analytikjena AAS Zeenit 700, Germany). Total carotenoids were calculated using the following formula and expressed as micrograms/g sample.

$$\text{Total}\;\text{carotenoids}=\frac{\text{Ab}\times V\times 10^{6}}{A^{1\%}}\times 100\times G,$$

where Ab is the absorbance at 470 nm, *V* is the total volume of extract, *A* ^1%^ is the extinction coefficient at 2500 for a 1% mixture of carotenoids, and *G* is the weight of the sample (g).

### 2.7 Determination of total phenolic and flavonoid contents

The melon pulp extract was prepared as described by Mallek‐Ayadi et al. (2018) and total phenolic compounds were determined according to Singleton and Rossi (1965). An aliquot of 0.5 ml of extract and 0.5 ml of Folin–Ciocalteu reagent were mixed. After 3 min incubation, 10 ml of sodium carbonate (75 g/L) was added and the mixture was incubated for 1 h. The absorbances were read using a spectrophotometer (Analytikjena AAS Zeenit 700) at 750 nm and the values were expressed as milligrams of gallic acid equivalents (GAE) per 100 g of fresh sample. Total flavonoid content was determined related to the colorimetric assay developed by Chang et al. (2002). An aliquot of 0.50 ml of properly diluted extract was mixed with 1.50 ml ethanol, 0.10 ml potassium acetate (1 M), 0.10 ml AlCl_3_ (10%), and 2.80 ml distilled water. The mixture was shaken vigorously and the absorbances were read at 415 nm using a spectrophotometer (Analytikjena AAS Zeenit 700). Results were expressed as milligrams of quercetin equivalents (QE) per 100 g of sample.

### 2.8 Quantification of phenolic compounds in melon pulp

Analyses of phenolic compounds in melon pulp extract were performed using a high‐performance liquid chromatography (HPLC) (Hewlett‐Packard System) system equipped with a HP‐1100 pump, a Rheodyne model 7725 injector (Cotati, CA, USA, loop volume 20 μl), a UV detector (280 nm), and a C_18_ Technochrom Eurosphere 100 analytical column (250 mm × 8 mm). An aliquot of 3 ml of Maazoun flesh extract was filtered using a 0.45‐μm membrane filter before injection. The HPLC separation was running for a total time of 24 min and the flow rate was maintained at 0.50 ml/min. The mobile phases for chromatographic analysis were: (A) acetonitrile and (B) sulfuric acid/water (2:98). A linear gradient was run from 15% (A) and 85% (B) to 40% (A) and 60% (B) during 12 min; it changed to 60% (A) and 40% (B) in 2 min; after 4 min, it changed to 80% (A) and 20% (B); and then to 90% (A) and 10% (B) after 2 min; it becomes 100% (A) during 4 min. The data were stored and processed by HPLC ChemStation (DOS Series) (Hewlett‐Packard). Cinnamic acid (purchased from Sigma‐Aldrich) was used as internal standard for the quantification of phenolic compounds and the results were expressed as milligrams of cinnamic acid per 100 g of flesh and as percentages.

### 2.9 Volatile compounds' extraction and identification

Melon cubes were juiced into a blender (Moulinex) and the volatiles' extraction was carried out by hydrodistillation for 6 h using a Clevenger‐type apparatus (Clevenger, 1928). Aroma volatiles were trapped in hexane and dried with anhydrous sodium sulfate. Then, volatiles' fraction was conserved at −20°C in amber glass vials hermetically closed until analysis. The gas chromatography (GC) analyses were performed using a Hewlett Packard HP‐5890 Series II instrument with dual flame ionization detector (FID) and equipped with DB‐WAX and DB‐5 capillary columns (30 m × 0.25 mm, 0.25‐μm film thickness), working with the following temperature program: 60–240°C at 3°C/min. Injector and detector temperatures were set at 220°C. The carrier gas was helium (2 ml/min) with a split ratio of 30:1. The identification of the components was performed, for the both columns, by comparison of their retention times with those of pure authentic samples and by mean of their linear retention indices (lri) relative to the series of *n*‐hydrocarbons.

Gas chromatography–mass spectrometry (GC–MS) analyses were accomplished with a Varian CP‐3800 gas chromatograph equipped with a DB‐5 capillary column (30 m × 0.25 mm; coating thickness 0.25 μm) and a Varian Saturn 2000 ion‐trap mass spectrometer. The injector and transfer line temperatures were set at 220°C and 240°C, respectively. The oven temperature was programmed from 60°C to 240°C at 3°C/min. Helium was used at 1 ml/min with a split ratio of 30:1. Identification of the volatile compounds was conducted by comparison between the retention times of the constituents and those of authentic samples, comparing also their linear retention indices relative to the series of *n*‐hydrocarbons, and on computer matching against commercial (Adams, 2017; NIST, 2014) and homemade library mass spectra built up from pure substances and components of known oils and mass spectrometry (MS) literature data (Adams, 2017). The amounts of the identified volatile compounds were expressed as relative abundance.

### 2.10 Tangential microfiltration of juice

**FIGURE 1:**
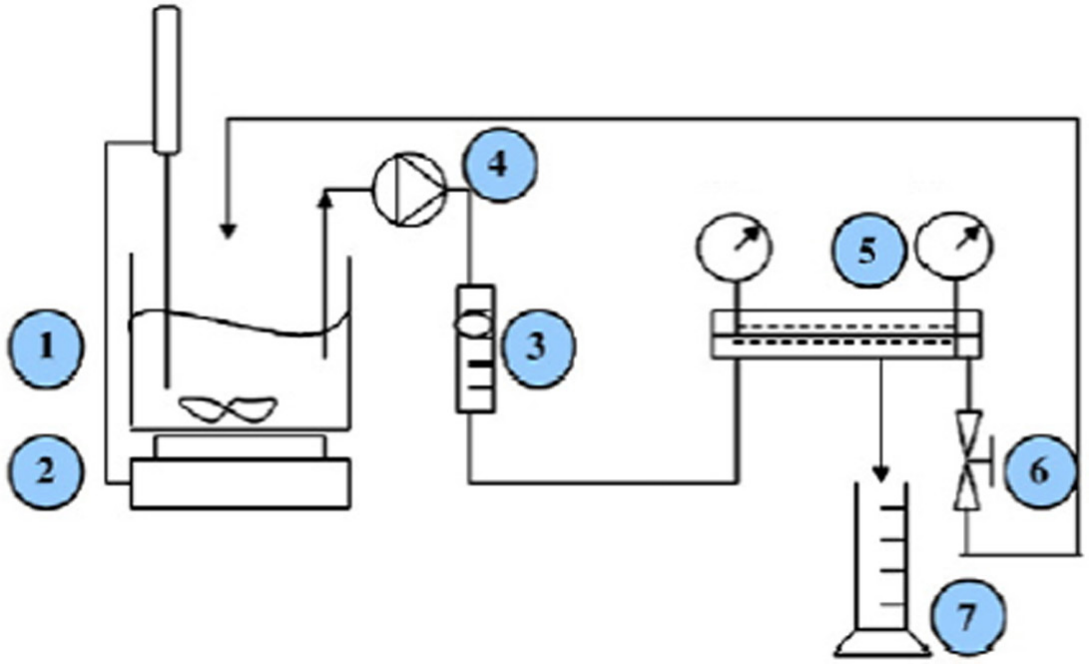
Scheme of the filtration process pilot. (1) Feed tank of capacity 2 L. (2) Regulated stirrer magnetic. (3) Flowmeter. (4) Feed pump. (5) Stainless steel membrane module. (6) Adjustable valve. (7) Test tube

The microfiltration tests were accomplished in a laboratory‐scale microfiltration unit incorporating monotubular membrane (CARBOSep) running in a crossflow mechanism with pressure control gauges. The mineral module has a total effective filtration area of 75 mm^2^ and an average pore diameter of 0.14 μm (M14 membrane). The microfiltration of melon juice was conducted at room temperature of 25 ± 1°C using the optimal process parameters which were established earlier: transmembrane pressure adjusted to 2 bars and tangential flow velocity of 0.91 L/m, with 2 L of the melon fruit sample in the feed tank. All results presented were an average of three experiments and the microfiltrations were carried out until the permeation flux remained almost constant. The juice microfiltration was performed in line with the batch concentration technique. According to this method, retentate was recycled to the feed tank and permeate was collected separately. A simplified scheme of the microfiltration equipment is shown in Figure 1. The system allowed the precise control of applied pressure and feed flow rate. Homogenization of the feed tank was assumed by the regulated stirrer magnetic. The permeate of each experiment was analyzed for pH, soluble solids (°Brix), titratable acidity, and color. After each experiment, the membrane module was cleaned in order to recover the original permeability. The pilot unit was rinsed with hot water (50°C). Then a 1% sodium hydroxide solution was recirculated (80°C/30 min with filtration). After rinsing with running water, a 0.5% nitric acid solution at 60°C was used during 30 min. Finally, the circuit board was rinsed with distilled water.

## 3 RESULTS AND DISCUSSION

### 3.1 Characterization of melon pulp

Physicochemical characteristics of fresh pulp from Maazoun variety are shown in Table 1. The moisture amount (83.05%) was similar to that reported by (Villanueva et al., 2004) for melon pulp from “Piel de Sapo” cultivar (83.60%). Because of its high water content, melon is a great snack during the hot summer months to prevent dehydration and to have refreshing sensation. The obtained results revealed that the lipid content (1.10%) was higher than those recognized for cantaloupe and watermelon pulps accounting for 0.28% and 0.15%, respectively (Fila et al., 2013; Li et al., 2006), nevertheless, lower than that of Brazilian melon flesh (1.80%; Morais et al., 2017). Moreover, Maazoun pulp contains 3.24% of proteins which was greater amount when compared to that of “Rochet” melon fruits having 1.14%, as mentioned by Villanueva et al. (2004). The ash content was higher (2.40 %) than that of cantaloupe pulp (0.71 %) related to Li et al. (2006).

**TABLE 1:** Proximate composition of *Cucumis melo* L. pulp (Maazoun variety)

Component	Value
Chemical composition (%)^a^	
Moisture	83.05 ± 0.10
Lipid	1.10 ± 0.05
Protein	3.24 ± 0.08
Ash	2.40 ± 0.04
Carbohydrate^b^	10.21 ± 0.12
Fiber	8.83 ± 0.09
Total soluble solids^c^	9.32 ± 0.07
pH	6.02 ± 0.01
Titratable acidity^d^	0.12 ± 0.02
Color	
L*	64.76 ± 0.05
a*	−3.58 ± 0.02
b*	7.94 ± 0.04
Total polyphenols^e^	206 ± 0.13
Total flavonoids^f^	71.46 ± 0.05
Minerals (mg/100 g)^g^	
Potassium	2113.75 ± 0.45
Magnesium	328.75 ± 0.11
Calcium	855.25 ± 0.19
Sodium	137.58 ± 0.72
Iron	1.82 ± 0.07
Zinc	0.7 ± 0.64
Manganese	0.48 ± 0.03
Copper	0.2 ± 0.02

Note

All determinations were carried out in triplicate and mean value ± standard deviation.

^a^ % of fresh weight.

^b^ Total carbohydrate obtained by difference.

^c^ °Brix.

^d^ Citric acid/100 g.

^e^ mg gallic acid equivalents (GAE)/100 g extract.

^f^ mg quercetin equivalents (QE)/100 g extract.

^g^ mg/100 g of dry matter.

Besides, the total carbohydrate amount, which accounted for 10.21%, was in accordance with that of honeydew flesh containing 9.18% (Li et al., 2006); however, the value was lower than that of melon pulp “Rochet” cultivar having 12.02%, as described by Villanueva et al. (2004). Generally, carbohydrates from food plants are often used for dietary purposes and are consequently beneficial for patient's health. Concerning dietary fiber, the total content mentioned in Table 1 was lower than that shown by Morais et al. (2017) for Brazilian melon flesh (8.83% vs. 12.20%). It can be assumed that melon, because of its water, carbohydrate, and dietary fiber contents, promotes regularity for a healthy digestive tract and helps therefore to prevent constipation problem (Kolayli et al., 2010). It is clear from these findings that Maazoun melon flesh has great potential to be used as a natural source of nutrients which are suitable as a nutraceutical supplement for producing functional foods.

### 3.2 Mineral composition of melon pulp

The mineral profile of melon pulp is detailed in Table 1. Potassium was the most abundant mineral studied in the edible part of Maazoun fruit containing 2113.75 mg/100 g followed by calcium accounting for 855.25 mg/100 g. In turn, magnesium concentration was 328.75 mg/100 g which represents the third important mineral element in melon pulp. Furthermore, sodium content was the lowest among analyzed macrominerals with an amount of 137.58 mg/100 g. These results were in agreement with other observations (Morais et al., 2017; Silva et al., 2018) reporting mineral compositions of melon fruits, displaying potassium and calcium as the major mineral elements. For comparison reason, the contents of calcium, magnesium, and sodium in Maazoun flesh were higher than those showed by Morais et al. (2017) for Brazilian melon pulp accounting for 165.30 mg/100 g, 130.90 mg/100 g, and 118.10 mg/100 g, respectively. Whereas, authors reported greater amount of potassium (3297.60 mg/100 g), when compared to our finding for this mineral element.

On the other hand, as compared to other fruits, it can be observed that the calcium content of melon pulp (855.25 mg/100 g) was nearly similar to that of fresh guavas' pulp (*Psidium guajava* L. var. Paluma) containing 815 mg/100 g (Pereira et al., 2009), but higher than that of the papaya pulp (“Golden” *Carica papaya* L.) having 267 mg/100 g, as reported by Oliveira et al. (2010).

Regarding microminerals, the highest amount was related to iron followed by zinc, manganese, and copper, whose values ranged from 0.20 to 1.82 mg/100 g, as can be seen in Table 1. The iron, zinc, manganese, and copper elements were found in relatively low levels for Maazoun melon in comparison to Brazilian melon whose amounts ranged between 1.00 and 4.10 mg/100 g (Morais et al., 2017). Even so, these minerals are essential, as they give information on the health status. Indeed, a number of microminerals such as manganese, copper, and zinc are cofactors of many enzymes. As well, iron is a component of several enzymes in the muscle cells (Spada et al., 2010). Based on the above results, it is noted that Maazoun flesh is rich in minerals, particularly potassium, magnesium, and calcium, which could be salutary for human nutrition.

### 3.3 Total soluble solids content, pH, and titratable acidity analyses

Melon maturity is determined on the basis of the sugar content, and as °Brix of the pulp. The TSS content is an important quality attribute of a fruit which is considered as an indicator of sugar amount related directly to the fruit sweetness. Maazoun melon exhibited similar TSS (9.32 °Brix; Table 1) to that of cantaloupe melon having 9.40 °Brix (Fundo et al., 2018). Moreover, (Solval et al., 2012) recorded a soluble solids content value of 10.57 °Brix for the cantaloupe variety. However, this value was considerably higher as compared to those of fresh‐cut Kırkağaç melons displaying 6.25 °Brix (Dilmaçünal et al., 2014). The minimum recommended sugar level for the melon fruits is 8 °Brix (Villanueva et al., 2004). Consequently, below that level, melons are not usually suitable for market.

The pH value (6.02) was similar to that of melon pulp “Piel de Sapo” cultivar having pH equal to 6.05, as assessed by Villanueva et al. (2004). It can also be observed that the Maazoun pH was in agreement with literature data (Dilmaçünal et al., 2014; Kolayli et al., 2010; Miller et al., 2017) for some melon varieties in which pH values were comprised to be between 5.61 and 7.09. As expected, the wide range of pH can be associated to the different species, geographical origin, climate factors, maturity stage, and cultural conditions. Regarding titratable acidity (TA), Maazoun pulp has 0.12 g citric acid/100 g which was significantly low when compared to the acidity of *cantaloupensis* melon having 0.92 g citric acid/100 g according to Güler et al. (2013).

### 3.4 Physical characteristics

#### 3.4.1 Pulp color

Melon flesh color is another important consumer acceptance attribute. CieLab coordinates of *C. melo* pulp are illustrated in Table 1. First, the lightness value was 64.76 through which the Maazoun pulp appeared bright, more vivid in color, and light colored when compared with that of cantaloupe melon having a brightness of 48.37, as described by Solval et al. (2012). Maazoun pulp was lighter colored and displayed thus bright color in comparison to the Italian melons flesh with 61.60 as L* value (Martuscelli et al., 2015). As can be clearly observed, the Maazoun flesh is visually a white‐greenish in color, as indicated by the negative a* parameter accounting for −3.58. This value was well matched with those reported by Oms‐Oliu et al. (2008) and Vanoli et al. (2015) for Spanish melons “Piel de Sapo” (−3.11) and “Honeydew Natal” cultivars (−3.0), respectively, which were characterized by a white‐greenish pulp. The b* parameter value of Maazoun pulp was low (7.94) when compared to that of Honeydew flesh (*Inodorus* variety) having 15.30 (Oms‐Oliu et al., 2008). Furthermore, the content of coloring components such as carotenoids and polyphenols was considered as attribute of the visual perception.

#### 3.4.2 Texture analysis

Firmness provides information about the rate of softening and the storage capacity of fruit. Fresh Maazoun pulp has a firm texture (14.81 N) as compared with cantaloupe (Güler et al., 2013) and *reticulatus* melon (Farcuh et al., 2020) pulps (9.10 N and 11.10 N, respectively). Compared with other fruits, the high firmness of pineapple fruit flesh accounted for 18.37 N (Sirijariyawat & Charoenrein, 2012) which revealed a soft texture of Maazoun melon pulp. The firmness texture attribute is associated with longer shelf‐life capacity of the fruit. According to Liu et al. (2019), firmness was typically used as a measure of eating quality, as well as an estimate of storability. Indeed, various industries applied puncture tests as part of their quality control procedure and fruit development to quantify desired characteristics. Additionally, some researchers (Liu et al., 2019; Vanoli et al., 2015) verified that fruit firmness is influenced by the species and cultivar. This fact could also be attributed to the genotypic effect and differences in physiological fruit maturity at various points in the fruit.

### 3.5 Vitamin C

Vitamin C is a powerful antioxidant found in numerous fruits, possessing a significant role in the suppression of free radicals, since it helps to prevent several human diseases associated with oxidative stress (Liu et al., 2015). The absolute value of vitamin C content of Maazoun melon pulp (31.50 mg/100 g) was high when compared to those of melon flesh (*Inodorus* variety) having 27.10 mg/100 g (Escribano & Lázaro, 2015) and watermelon pulp accounting for 9.39 mg/100 g (Johnson et al., 2013). Moreover, Liu et al. (2015) evaluated the vitamin C content in “Wulingyulu” peach pulp and reported a concentration of 5.54 mg/100 g. It was clear from these results that, melon fruits exhibited high ascorbic acid amounts as compared to peaches. Whereas, the vitamin C content of Maazoun melon was lower than that of cantaloupe melon having 88.08 mg/100 g (Fundo et al., 2018). Overall, the Maazoun pulp could be considered a substantial source of vitamin C and thus used as a natural ingredient to develop novel food products with high nutritional and antioxidant properties.

### 3.6 Carotenoid content

Carotenoids are considered as bioactive molecules whose consumption is related to health benefits. The quantitative measurement of the carotenoids in melon pulp may provide information to support its use as a natural source of biologically active compounds. The total carotenoids' concentration of Maazoun pulp (41.72 µg/g) was lower when compared to that of *C. melo* L. *reticulatus* variety accounting for 68.92 µg/g (Fundo et al., 2018). Another work developed by Kandlakunta et al. (2008) on pumpkin (*Cucurbita maxima* yellow cultivar) revealed that the pulp has a carotenoid content of 21.20 μg/g. Interestingly, carotenoids exhibit antioxidant properties and act as a scavenger of free radicals with potential beneficial effects on human health (Silva et al., 2018).

### 3.7 Total phenolic and flavonoid contents of melon pulp

Polyphenols are the most abundant antioxidants in diets based on fruits and vegetables, and they are known as nutrients for human health. The total phenolic content assessed in Maazoun pulp (206 mg/100 g) was higher than those obtained for cantaloupe flesh having 168 mg/100 g (Ismail et al., 2010) and “Wulingyulu” peach pulp accounting for 86.33 mg/100 g (Liu et al., 2015), while, the total phenolic content of Maazoun pulp was lower by comparison with those of Italian melon flesh (Martuscelli et al., 2015) and mango pulp (Silva et al., 2014) displaying 379 mg/100 g and 652.59 mg/100 g, respectively. On the other hand, Maazoun melon flesh showed lower total phenolic compounds than Maazoun peels (206 vs. 332 mg/100 g) (Mallek‐Ayadi et al., 2017). A similar trend was ascertained for many fruits such as pomegranate, mango, and apple, in which peels contained more polyphenols than fleshes, and exhibited good antioxidant and antiproliferative activities (Li et al., 2013). It should be emphasized that the phenolic phytochemicals have the capacity to protect cellular components against free radicals *via* their antioxidant and free radical scavenging effects (Kim et al., 2010).

Flavonoids are recognized as strong antioxidant agents possessing a substantial role in the prevention of diseases and human protection. The total flavonoid content of Maazoun pulp (71.94 mg/100 g) was in agreement with that of Brazilian melon flesh (72.62 mg/100 g) as recorded by (Morais et al., 2015). It was also found that the total flavonoid content in Maazoun flesh was quite higher as compared to that in watermelon pulp accounting for 36.81 mg/100 g (Morais et al., 2015). Considering mentioned data, it was possible to verify that Maazoun melon pulp has considerable amounts of phenolic compounds and this suggests that melon can be a cheap source of biologically active phytochemicals with potential industrial applications.

### 3.8 Identification and quantification of phenolic compounds by HPLC

**FIGURE 2:**
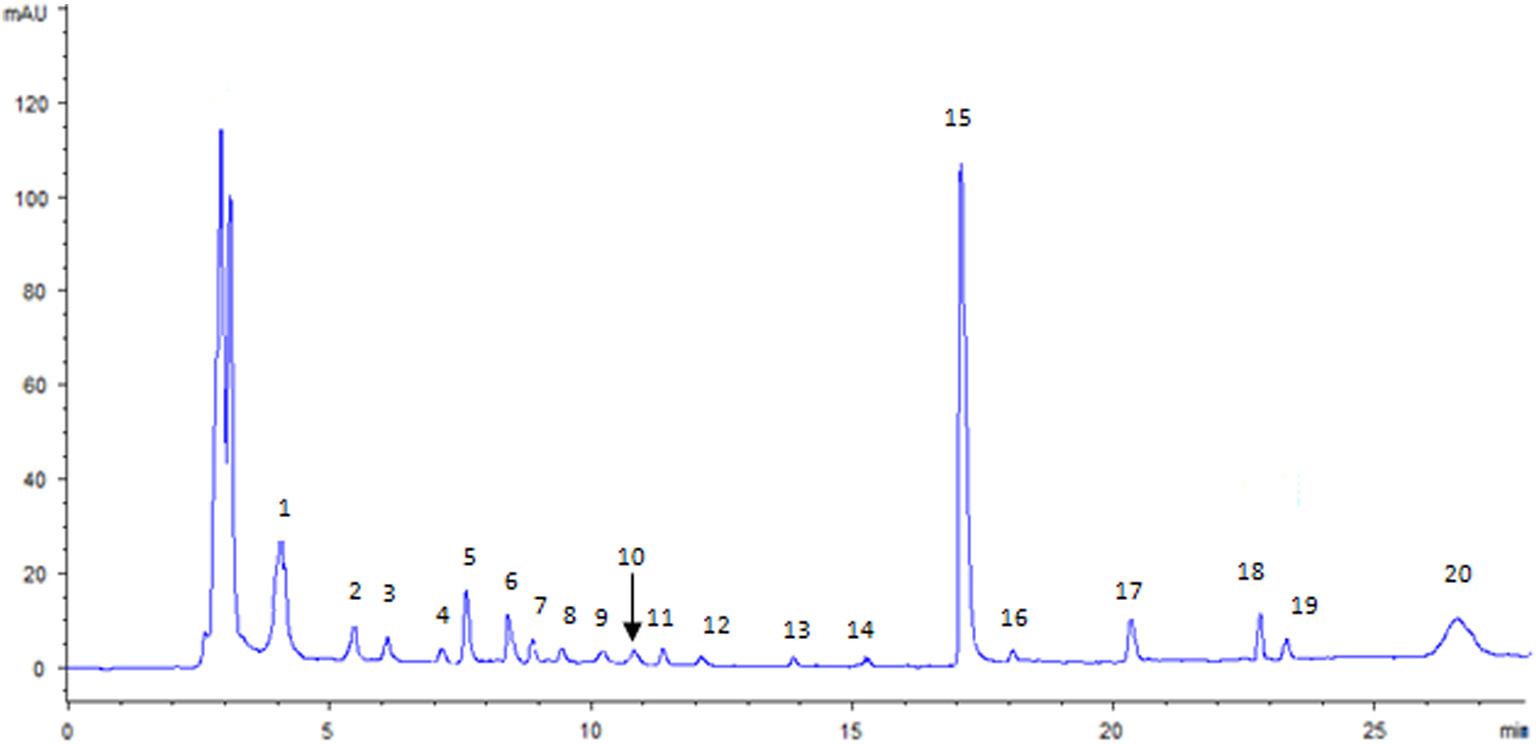
High‐performance liquid‐chromatography (HPLC) chromatogram of phenolic compounds in melon pulp extract of Maazoun cultivar. Detection was at 280 nm. Peak: (1) Gallic acid, (2) hydroxytyrosol, (3) protocatechuic acid, (4) chlorogenic acid, (5) 4‐hydroxybenzoic acid, (6) isovanillic acid, (7) 4‐hydroxybenzoic acid, (8) luteolin‐7‐glycoside, (9) *p‐*coumaric acid, (10) apigenin‐7‐glycoside, (11) oleuropein, (12) *m‐*coumaric acid, (13) phenylacetic acid, (14) luteolin, (15) cinnamic acid (IS=internal standard), (16) apigenin, (17) flavone, (18) unknown, (19) isoflavone, and (20) amentoflavone

To the best of our knowledge, data regarding phenolic composition of melon flesh were very scarce in the literature, since the available information involves uniquely the quantification of total phenolic compounds and total flavonoid contents as described by some researchers (Ismail et al., 2010; Kolayli et al., 2010; Morais et al., 2015). The HPLC analysis of the phenolic fraction of Maazoun pulp revealed a total of 20 individual compounds. The HPLC chromatogram of phenolic compounds is given in Figure 2. Melon pulp polyphenols belong to diverse chemical classes: nine phenolic acids, seven flavonoids, one phenolic monoterpene, and one secoiridoid. Interestingly, both phenolic acids and flavonoids were abundant in Maazoun melon pulp as seen in Table 2. Indeed, phenolic acids constitute the main class of phenolic phytochemicals (27.57 mg/100 g) followed by flavonoids (24.34 mg/100 g). This trend was also observed for melon peels and melon seed oil (Mallek‐Ayadi et al., 2017, 2018) in which flavonoids and phenolic acids were the major groups of phenolic compounds.

**TABLE 2:** Contents (mg/100 g) and percentages (%) of phenolic compounds of Maazoun melon (*Cucumis melo* L.) pulp

Phenolic compounds	Content	Percentage
Phenolic acids	27.57 ± 0.28	47.52
Gallic acid	13.56 ± 0.08	23.43
Protocatechuic acid	1.54 ± 0.03	2.56
Chlorogenic acid	1.01 ± 0.01	1.75
4‐Hydroxybenzoic acid	3.84 ± 0.02	6.64
Isovanillic acid	2.63 ± 0.01	4.54
3‐Hydroxybenzoic acid	1.86 ± 0.00	3.21
*p*‐Coumaric acid	1.31 ± 0.01	2.24
*m*‐Coumaric acid	0.92 ± 0.04	1.58
Phenylacetic acid	0.91 ± 0.03	1.57
Flavonoids	24.34 ± 0.15	42.57
Luteolin‐7‐glycoside	1.14 ± 0.00	1.93
Apigenin‐7‐glycoside	1.43 ± 0.02	2.42
Luteolin	0.57 ± 0.04	0.98
Apigenin	0.90 ± 0.03	1.54
Flavone	2.49 ± 0.07	4.29
Isoflavone	1.68 ± 0.05	3.51
Amentoflavone	16.14 ± 0.14	27.90
Phenolic monoterpene	2.48 ± 0.06	4.26
Hydroxytyrosol	2.48 ± 0.06	4.26
Secoiridoid	1.37 ± 0.09	2.30
Oleuropein	1.37 ± 0.09	2.30
Unknown	2.03 ± 0.06	3.43
Total	57.80 ± 0.74	100

Note

All determinations were carried out in triplicate and mean value ± standard deviation.

In the present work, amentoflavone and gallic acid were the major phenolic compounds in melon pulp having 16.14 mg/100 g and 13.56 mg/100 g, respectively. Looking at these compounds, it was crucial to mention that amentoflavone was known as the potent antioxidant and anti‐inflammatory agent (Fundo et al., 2018). Furthermore, gallic acid has been widely used as additive to prevent rancidity of foods and it also exhibits anti‐inflammatory activity (Silva et al., 2018). This phenolic acid was found to be the major phenolic acid in bitter melon pulp accounting for 9.23 mg/100 g (Lee et al., 2017) and the second main phenolic acid identified for melon peel as verified by Mallek‐Ayadi et al. (2017), nevertheless, Maazoun melon peel presented a lower amount of gallic acid (12.07 mg/100 g). Furthermore, as previously described by Kolayli et al. (2010), the analysis of phenolic compounds of melon fruit exhibited 14 phenolic acids in which benzoic acid was the principal component found (30.06 mg/100 g), followed by abscisic acid (15.39 mg/100 g) and vanillic acid (7.24 mg/100 g). In turn, gallic acid was found in a small amount of 1.74 mg/100 g.

On the other hand, melon flesh extract contains notable amounts of 4‐hydroxybenzoic acid (3.84 mg/100 g), isovanillic acid (2.63 mg/100 g), and flavones (2.49 mg/100 g). It is interesting to note that isovanillic acid and 4‐hydroxybenzoic acid possess antimicrobial and antioxidant properties (Khadem & Marles, 2010). Bataglion et al. (2015) asserted that flavonoids and phenolic acids were considered as the major bioactive molecules found in fruits, displaying potential natural antioxidant properties. Other phenolic compounds, such as hydroxytyrosol, 3‐hydroxybenzoic acid, isoflavone, and apigenin‐7‐glycoside, were also identified in Maazoun melon pulp with amounts of 2.48, 1.86, 1.68, and 1.43 mg/100 g, respectively. It is worth noting that apigenin‐7‐glucoside has attracted attention for its antiproliferative and antitumoral effects (Silva et al., 2018). The above results demonstrated that melon pulp contained several biologically active compounds of which certain of them present antitumor potential (Weng & Yen, 2012). Thereby, this fruit may have positive effects on health and can be used as a food supplement for new industrial purposes.

### 3.9 Volatile composition of Maazoun melon

In order to investigate the aroma properties of Maazoun melon, relative volatile concentrations were determined and altogether 38 volatile components were thus detected. As can be observed from Table 3, esters represent the major volatile class, comprising more than 70% of total measured volatiles. The headspace of Maazoun flesh was dominated by ethyl acetate (18.08%), 2‐methylpropyl acetate (15.76%), and 2‐methylbutyl acetate (12.71%), which were the principal compounds, with an array of small percentages of butyl acetate, butyl propanoate, ethyl butanoate, and hexadecanoic acid‐methyl ester. The same trend was observed for several melon cultivars including Galia (Lignou et al., 2013), Navigator (Vallone et al., 2013), and Charentais (Lignou et al., 2014) in which ester compounds were predominant. Similarly, in a study conducted by Allwood et al. (2014) on five different melon cultivars, it has been evidenced that esters represented the principal family of detected volatiles. Interestingly, the melon aroma is mainly associated to the components of greatest abundance. Hence, the “fruity” and “pulpy” aromas' sensory attributes of the melon pulp from Maazoun variety were significantly correlated with esters group. For instance, butyl acetate, which was frequently quantified with high amounts in melons, conveys “floral” note and ethyl 3‐(methylthio) propanoate describes “melon/fresh/green” characteristics of melon aroma (Amaro et al., 2012; Vallone et al., 2013). On the other side, researchers opined that aroma of some ripe fruits, such as melons, Asian pears, and pineapples, was due to the presence of esters (Allwood et al., 2014).

**TABLE 3:** Relative percentages of volatile compounds in melon pulp (Maazoun cultivar)

No.	Name	CAS number^a^	LRI^b^	Relative peak area (%)
	**Esters**			
1	Ethyl acetate	0141‐78‐6	619	18.08 ± 0.22
2	Isobutyl acetate	0110‐19‐0	775	2.34 ± 0.02
3	2‐Methylpropyl acetate	0110‐19‐0	776	15.76 ± 0.15
4	Ethyl butanoate	0105‐54‐4	804	3.46 ± 0.10
5	Butyl acetate	0123‐86‐4	816	5.24 ± 0.03
6	2‐Methylbutyl acetate	0624‐41‐9	880	12.71 ± 0.11
7	Ethyl pentanoate	0539‐82‐2	903	0.19 ± 0.01
8	Butyl propanoate	0590‐01‐2	910	5.32 ± 0.01
9	Methyl hexanoate	0106‐70‐7	925	2.06 ± 0.04
10	Ethyl hexanoate	0123‐66‐0	999	1.70 ± 0.07
11	Heptyl acetate	0112‐06‐1	1113	0.20 ± 0.05
12	Benzyl acetate	0101‐41‐7	1164	0.44 ± 0.01
13	9‐Octadecenoic acid (Z)‐, methyl ester	0112‐62‐9	2096	1.60 ± 0.01
14	Hexadecanoic acid, methyl ester	0112‐39‐0	2158	2.39 ± 0.03
15	Nonanoic acid, methyl ester	1731‐84‐6	2172	0.62 ± 0.02
16	Hexadecanoic acid	0057‐10‐3	2886	1.49 ± 0.01
	% Identified			73.60 ± 0.24
	**Sulfur‐containing compounds**			
17	Ethyl‐(methylthio) acetate	04455‐13‐4	984	1.19 ± 0.02
18	Ethyl 3‐(methylthio) propanoate	13327‐56‐5	1099	1.47 ± 0.05
	% Identified			2.66 ± 0.11
	**Alcohols**			
19	2‐Methylpropanol	10143‐32‐5	633	1.1 ± 0.04
20	2‐Methylbutanol	02461‐18‐9	749	1.23 ± 0.01
21	1‐Hexanol	00111‐27‐3	872	2.76 ± 0.05
22	Myrcenol	00543‐39‐5	1119	0.97 ± 0.01
23	3‐Nonen‐1‐ol	10340‐23‐5	1157	5.09 ± 0.12
24	Pulegol	22472‐80‐6	1220	1.32 ± 0.02
25	*p*‐Menth‐8(10)‐en‐9‐ol, trans‐	15714‐12‐2	1224	0.20 ± 0.01
26	(2Z,6E)‐Farnesol	03790‐71‐4	1704	0.19 ± 0.01
27	(2E,6E)‐Farnesol	00106‐28‐5	1709	0.21 ± 0.01
	% Identified			13.07 ± 0.61
	**Aldehydes**			
28	2‐Methylbutanal	00096‐17‐3	660	0.23 ± 0.01
29	Hexanal	00066‐25‐1	802	0.83 ± 0.02
30	(E)‐2‐Nonenal	18829‐56‐6	1159	0.64 ± 0.01
31	Decanal	00112‐31‐2	1205	0.22 ± 0.03
	% Identified			1.92 ± 0.21
	**Terpenoids**			
32	Limonene	5989‐27‐5	1026	0.71 ± 0.01
33	Eucalyptol	0470‐82‐6	1041	1.34 ± 0.05
34	β‐Caryophyllene	0087‐44‐5	1437	0.75 ± 0.02
35	Phytol	0150‐86‐7	2099	3.58 ± 0.06
	% Identified			6.38 ± 0.21
	**Acids**			
36	9‐Octadecenoic acid (Z)‐	0112‐80‐1	2133	0.83 ± 0.03
37	9,12‐Octadecadienoic acid (Z,Z)‐	0060‐33‐3	2144	1.12 ± 0.01
	% Identified			1.95 ± 0.80
	**Carbonyl compound**			
38	β‐Cyclocitral	0432‐25‐7	1218	0.42 ± 0.03
	% Identified			0.42 ± 0.03
	**% Total identified**			100 ± 0.86

Note

Results are expressed as means ± standard deviations (*n* = 3).

^a^ Chemical Abstracts Service.

^b^ Linear retention indices (DB‐5 column).

Moreover, quantitative distribution of volatile compounds in Maazoun pulp indicated the presence of sulfur compounds, among them ethyl‐(methylthio) acetate and ethyl 3‐(methylthio) propanoate which contribute actively to the aroma profile of melons (Amaro et al., 2012). Likewise, Lignou et al. (2014) reported some results for melon aromas, concluding that the “musky” note characteristic is arranged by sulfur compounds' fraction of this fruit because it constitutes potent odorants.

In addition to esters and sulfur‐containing compounds, numerous alcohols, terpenoids, and aldehydes were detected in the samples. In fact, alcohols were identified as the second major family of volatiles recovered in fresh melons. Quantitatively, as listed in Table 3, the 3‐nonen‐1‐ol was recognized to be the principal alcohol identified, which describes “melon/fresh/green” notes. Lignou et al. (2014) arrived at similar conclusions when studying volatiles' content in sweet melon pulp from Galia cultivar. These authors reported that the 3‐nonen‐1‐ol presented the highest amount when compared to the levels of identified alcohols. Furthermore, it was worth mentioning that the *p*‐menth‐8(10)‐en‐9‐ol was characterized for the first time in melons. In the current study, it was observed that the volatile profile was consistent with the published volatile fraction compositions of Galia and Charentais melons in which esters and alcohols presented, respectively, the first and second classes (Lignou et al., 2013, 2014). Furthermore, the volatile profile analyzed by GC–MS was found to possess terpenoids like phytol, eucalyptol, and β‐caryophyllene representing approximately 6% of the total volatiles. The eucalyptol content in Maazoun pulp (1.34%) was low as compared to that in melon flesh from *reticulatus* variety (3.88%), as described by Amaro et al. (2012). Furthermore, eucalyptol, which imparts “sweet” and “fruity” notes, demonstrated antimicrobial and anti‐inflammatory properties. Yet, phytol compound was reported to play antioxidant effect and β‐caryophyllene was supposed to have anti‐inflammatory and anticancer activities (Peng et al., 2019).

The results also showed that a series of volatiles belonging to a chemical class of aldehydes was identified (Table 3) and was found to be less abundant than esters, alcohols, and terpenoids. As expected, aldehydes serve as key aroma compounds in fruits where they impart “green” note and “grassy” description. Aldehydes have been shown to possess important levels in immature melon fruits, contrariwise to esters which were abundant in ripe melons, as stated above.

More importantly, a better understanding of the volatile profile of Maazoun fruit could attract great interest due to its aroma richness and unique volatiles' composition. Hence, Maazoun pulp could be used as a juice and/or as a flavoring and coloring agent in food products for better consumer attractiveness.

### 3.10 Microfiltration of juice

#### 3.10.1 Flux evaluation

**FIGURE 3:**
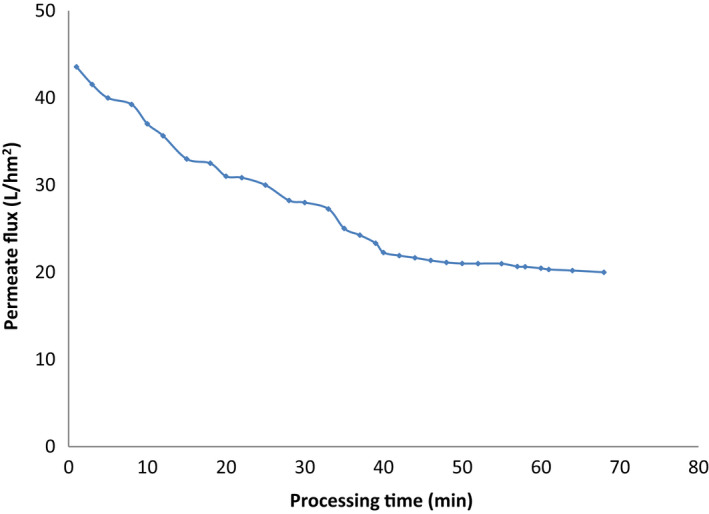
Permeate flux profile during microfiltration of melon juice from Maazoun cultivar

A melon juice was filtered using a crossflow membrane filtration through M14 membrane in order to remove suspended solids and to obtain a clear juice. Figure 3 shows the evolution of the juice permeate flux with operating time at constant value of temperature (25°C ± 1°C), transmembrane pressure (2 bars), and flow rate (0.91 L/min). Based on the graph, drastic declination of permeate flux of Maazoun juice can be seen at the initial period followed by a gradual reduction after this period. Indeed, the flux trajectory comprises two distinct zones. These observations corroborate the results for the clarification of prickly pear juice (Ennouri et al., 2006), pomegranate juice (Cassano et al., 2011), lemon juice (Maktouf et al., 2014), and guava juice (Omar et al., 2019). The initial permeate flux of the M14 membrane (around 44 L/h/m^2^) decreased and tended to stabilize, until it reached a steady state of about 20 L/h/m^2^. A rapid flux decline was noticed with a reduction of more than 50% during the first 40 min, followed by a relatively low flux. For comparison reason, a similar stabilized flux attaining 19.75 L/h/m^2^ has been observed by Espamer et al. (2006) for the clarification of lemon fruit juice. Furthermore, the operating time used to obtain a 50% reduction in the permeation flux of melon juice is less than that obtained for the reduction to 50% of the flux during clarification by microfiltration of the prickly pear juice which is equal to 60 min (Ennouri et al., 2006). These trends were strongly related to the fouling phenomenon that occurred on the membrane surface during the filtration process. This phenomenon designated by concentration polarization frequently means flux decline and decrease of microfiltration performance. Subsequently, when the feed passed through the membrane and left suspended solids behind, the rejected particles started to deposit on the membrane surfaces to create fouling (Castro‐Muñoz et al., 2018). That's why, it is crucial to mention that the permeate flux is a function of time and depends on operating conditions, nature of the membrane, and type of the feed solution.

#### 3.10.2 Quality of filtered juice

Since the separation process is nonthermal and involves a minimal loss of nutrients, membrane processes are recently used in various productive sectors (Cassano et al., 2020). Table 4 reports the physicochemical properties of the feed and permeate of the separation membrane process at selected operating conditions. It was evident from this table that the effects of the microfiltration process on juice quality parameters were insignificant. The light decrease in TSS compared to feed was caused by the removal of suspended solids. In fact, the microfiltration was quite successful in removing suspended solids from the freshly produced melon juice. Refractometer readings of the TSS content on permeate were lower when compared to those for the feed. This aspect can be explained by the fact that there was a high amount of suspended solids which was rejected by the membrane along with the pulp (Cassano et al., 2007), although there was only a slight reduction of TSS in permeate. There was a remarkable improvement in the clarity of melon juice after filtration because of the removal of suspended colloidal particles present in the raw juice. The literature has reported similar findings in the juice clarification of prickly pear (Castro‐Muñoz et al., 2018) and kiwifruit (Cassano et al., 2007). The TSS generally consisted of low‐molecular‐weight compounds such as sugars (sucrose, glucose, and fructose), minerals, salts, and some organic acids (Castro‐Muñoz et al., 2018). Therefore, almost all the sugars and acids present in the feed can be recovered in permeate after filtration. The acid is also mostly recovered in permeate. Besides, the clarification process did not have much effect on the pH of the juice. Indeed, microfiltered melon juice is slightly more acidic (5.42) than raw juice (5.50). This can be explained by the presence of soluble organic acids in permeate.

**TABLE 4:** Physicochemical properties of raw melon juice (feed) and filtered juice (permeate) from Maazoun cultivar

Parameter	Feed	Permeate
Total soluble solids	9.32 ± 0.02	8.46 ± 0.02
pH	6.02 ± 0.02	5.94 ± 0.02
Titratable acidity	0.12 ± 0.02	0.10 ± 0.02
Color		
L*	59.10 ± 0.02	64.76 ± 0.07
a*	−3.33 ± 0.05	−0.40 ± 0.12
b*	30.46 ± 0.02	36.37 ± 0.03

Note

All values given are means of three determinations.

L*: lightness; a*: red‐green dimension; b*: yellow‐blue dimension.

With respect to luminosity, all permeate samples were light in color as they possessed higher L* value (64.76 vs. 59.10) showing that clarified juice was more translucent than the raw juice. The increase in the permeate brightness compared to that of the feed was also noticed by Ennouri et al. (2015) who reported that the value of L* parameter increases from 46.6 to 65 following the microfiltration of carrot juice. Furthermore, the increase of a* component of permeate (−0.40) underlines that the microfiltered juice has lost few of green color when compared to that of raw melon juice while remaining negative (green tint). Moreover, the b* parameter value of the permeate (36.37) was higher than that of the raw melon juice (30.46) which gave the juice an accented yellow color. This can be attributed to the molecules' depletion in the filtered juice that affects the juice color such as phenolics and pigments. According to Labaky et al. (2020), the increase of a* and b* reveals biochemical changes (formation of carotenoids, chlorophyll degradation, etc.). Thus, the color analysis put in evidence the loss of green color and the accentuation of yellow color. Interestingly, the introduction of separation process technologies in the industrial transformation cycle of melon juice represents one of the technological solutions to obtain a quality, natural fresh taste, and additive‐free product.

## 4 CONCLUSION

Within this study, melon flesh from Maazoun cultivar contains phytochemicals and micronutrients with great nutritional potential. The pulp was also found to be a cheap source of bioactive molecules like phenolic acids and flavonoids, which are natural antioxidants that possess beneficial health effects. Regarding volatile profile, esters family appeared to be the essential contributor to the desirable aroma of Maazoun fruit. The clarification of melon juice showed that microfiltration can clarify melon juice without causing significant changes in its physicochemical characteristics. Maazoun melon pulp could be considered as a fruit with positive nutritional value and bioactive effects. These results are of crucial interest since *C. melo* has a good appreciation by consumers. In addition, melon fruit pulps could have a potential use to isolate specific phytochemicals for application in nutraceutical supplements, dietary additives, and new food products.

## CONFLICT OF INTEREST

The authors declare that there is no conflict of interests.

## AUTHOR CONTRIBUTIONS

**Sana Mallek‐Ayadi:** Conceptualization (equal); Data curation (equal); Formal analysis (equal); Investigation (equal); Methodology (equal); Software (equal); Writing – original draft (equal); Writing – review & editing (equal). **Neila Bahloul:** Methodology (equal); Software (equal); Visualization (equal). **Semia Baklouti:** Methodology (equal); Supervision (equal); Validation (equal). **Nabil Kechaou:** Funding acquisition (equal); Project administration (equal); Resources (equal); Supervision (equal).

## Data Availability

Data sharing not applicable to this article as no datasets were generated or analysed during the current study.
